# Supplementary material for: p63 is a key regulator of iRHOM2 signalling in the keratinocyte stress response
Source: Nat Commun. 2018 Mar 9;9:1021. doi: 10.1038/s41467-018-03470-y (PMC5844915; doi:10.1038/s41467-018-03470-y)
Supplement: Supplementary file 1 — Supplementary Information [file 41467_2018_3470_MOESM1_ESM.pdf]

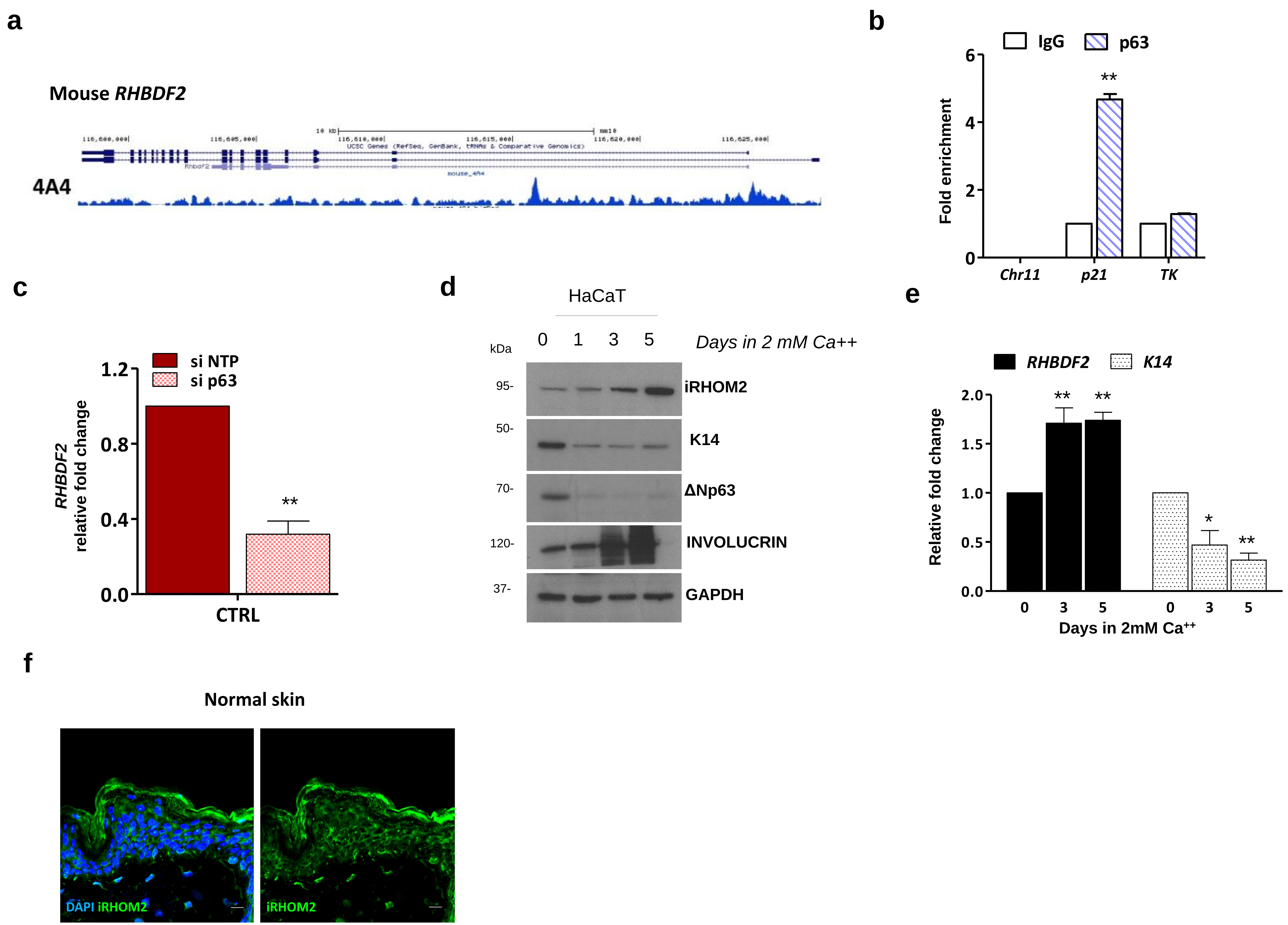
a
Mouse RHBDF2
4A4
b
IgG
p63
Fold enrichment
6
4
2
0
**
Chr11
p21
TK
c
si NTP
si p63
RHBDF2 relative fold change
1.2
0.8
0.4
0.0
**
CTRL
d
HaCaT
0 1 3 5
Days in 2 mM Ca++
kDa
95-
50-
70-
120-
37-
iRHOM2
K14
ΔNp63
INVOLUCRIN
GAPDH
e
RHBDF2
K14
Relative fold change
2.0
1.5
1.0
0.5
0.0
**
**
*
**
0 3 5 0 3 5
Days in 2mM Ca++
f
Normal skin
DAPI iRHOM2
iRHOM2

Supplementary Figure 1. Identification of iRHOM2 as a p63 target gene in keratinocytes. (a) Screenshot of the UCSC genome browser from ChIP-seq analysis of normal mouse primary keratinocytes with 4A4 antibody. The ChIP-seq study was previously reported. **(b)** ChIP-qPCR analysis for the specific binding of p63 was performed with a no-gene region (Chr11), *p21* (positive control) and Thymidine kinase (*TK*) (negative control), with α-pan-p63 (H137) and α-IgG antibodies in CTRL cells. **(c)** qRT-PCR of *RHBDF2* in CTRL keratinocytes transfected with NTP or p63 siRNA. The graph represents means and SEM of three biological replicates. Student's t-test was performed ($p < 0.01$(**)). **(d)** Immunoblotting shows induction of differentiation in HaCaT cells by treatment with 2 mM calcium for five days. Expression of iRHOM2, K14, INVOLUCRIN, ΔNp63 were analysed at the time points indicated. GAPDH was used as a loading control. **(e)** qRT-RCR for *RHBDF2* and *K14* was performed in HaCaT cells treated with calcium for three and five days. The graph represents mean with SEM of four independent experiments. Student's t-test was used for statistical evaluation ($p<0.05$ (*) and $p<0.01$ (**)). **(f)** Confocal analysis of iRHOM2 in control interfollicular skin. DAPI (blue) is used as a nuclear stain. Scale bar: 20 µm**.**

Supplementary Fig. 2

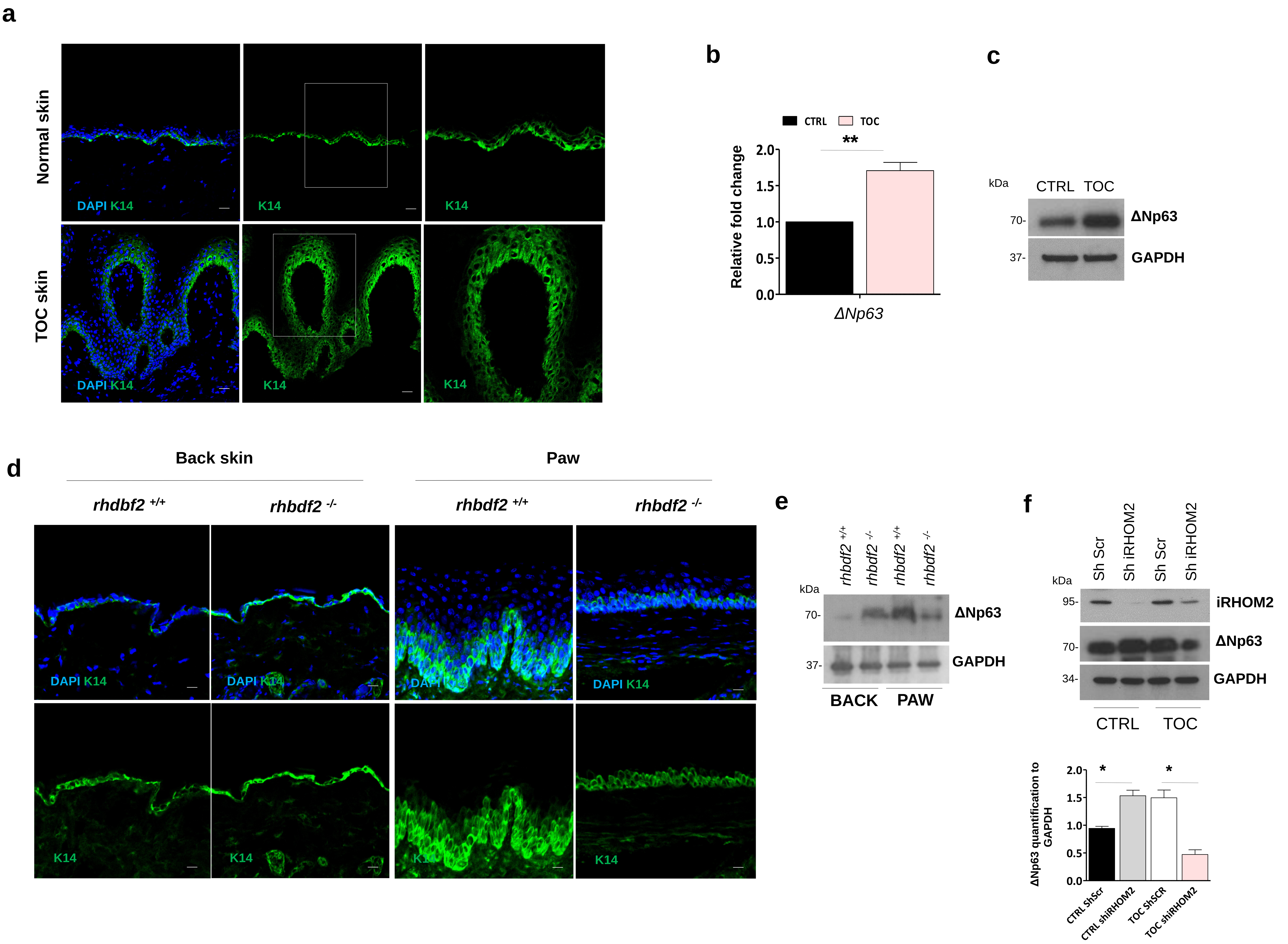

Supplementary Figure 2. Distinct regulation of p63 in normal and hyperproliferative keratinocytes. (a) Representative images showing K14 expression in TOC epidermis and in control interfollicular skin. DAPI (blue) is used as a nuclear stain. Scale bars: 20 µM. **(b)** Relative mRNA levels of *ΔNp63* in control (CTRL) and TOC keratinocytes are shown. Data represent three independent experiments with similar results. For statistical analysis, Student's *t*-test was used, comparing TOC with CTRL cells. **(c)** Western blotting analysis for ΔNp63 expression in CTRL and TOC keratinocytes, with GAPDH as loading control. **(d)** Representative confocal analysis of K14 expression was performed in back skin and fore-paw sections from *rhbdf2*$^{+/+}$ and *rhbdf2*$^{-/-}$ mice. DAPI (blue) is used as a nuclear stain. Scale bar: 20 µm. **(e)** Representative WB for ΔNp63 in the back skin and fore-paw of *rhbdf2*$^{+/+}$ and *rhbdf2*$^{-/-}$ mice. **(f)** Expression of ΔNp63 by WB in CTRL and TOC keratinocytes depleted for iRHOM2. GAPDH was used as a loading control. The quantification included was performed by ImageJ software in comparison to the loading control in three independent experiments. Error bars represent SEM and Student's two-tailed t-test values are given ($p < 0.05$ (*)).

Supplementary Fig. 3

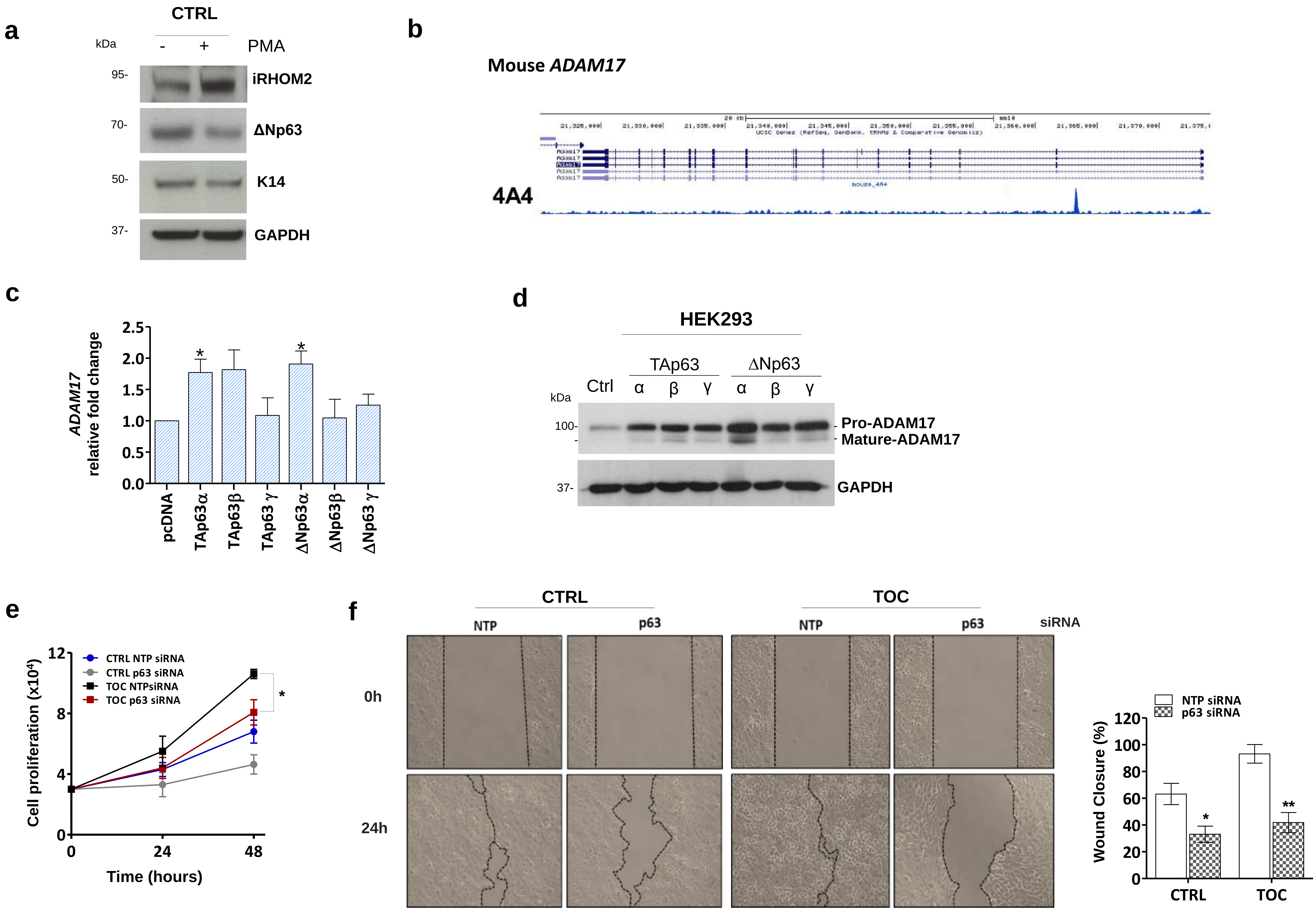

Supplementary Figure 3. iRHOM2-ADAM17 axis regulates p63 expression. (a) Expression levels of iRHOM2, ΔNp63 and K14 by WB in CTRL keratinocytes after treatment with PMA (250 ng/ml) for 24 hours. GAPDH was used as a loading control. **(b)** Screenshot of the UCSC genome browser from ChIP-seq analysis of normal mouse primary keratinocytes with 4A4 antibody. The p63 binding sites identified in this ChIP-seq study were previously reported. **(c)** qRT-PCR was performed for *ADAM17* in HEK293 cells transfected with TA and ΔNp63 isoforms, 9 hours post transfection. The graph represents means and SEM of three biological replicates. Student's two-tailed t-test was used for statistical evaluation ($p<0.05$ (*)). **(d)** Representative WB for ADAM17 was performed in HEK293 cells over-expressing p63 isoforms. GAPDH was used as loading control. **(e)** Growth curves of cultured CTRL and TOC keratinocytes transfected with non-targeting pool (NTP) or p63 siRNA at 0, 24 and 48 hours. Data are expressed as mean $\pm$ SEM. Student's t-test was performed comparing NTP and p63 siRNA treatment in each cell line at the indicated time point ($p<0.05$ (*)). **(f)** Representative images from scratch assay at 0 and 24 hours, in CTRL and TOC keratinocytes transfected with NTP or p63 siRNA, using a phase-contrast microscope. Quantification of the wound closure was analysed using by ImageJ . The graph represents the mean of three experiments. Error bars denote $\pm$ SEM. Student's two tailed t-test shows $p<0.05$ (*) and $p<0.01$ (**).

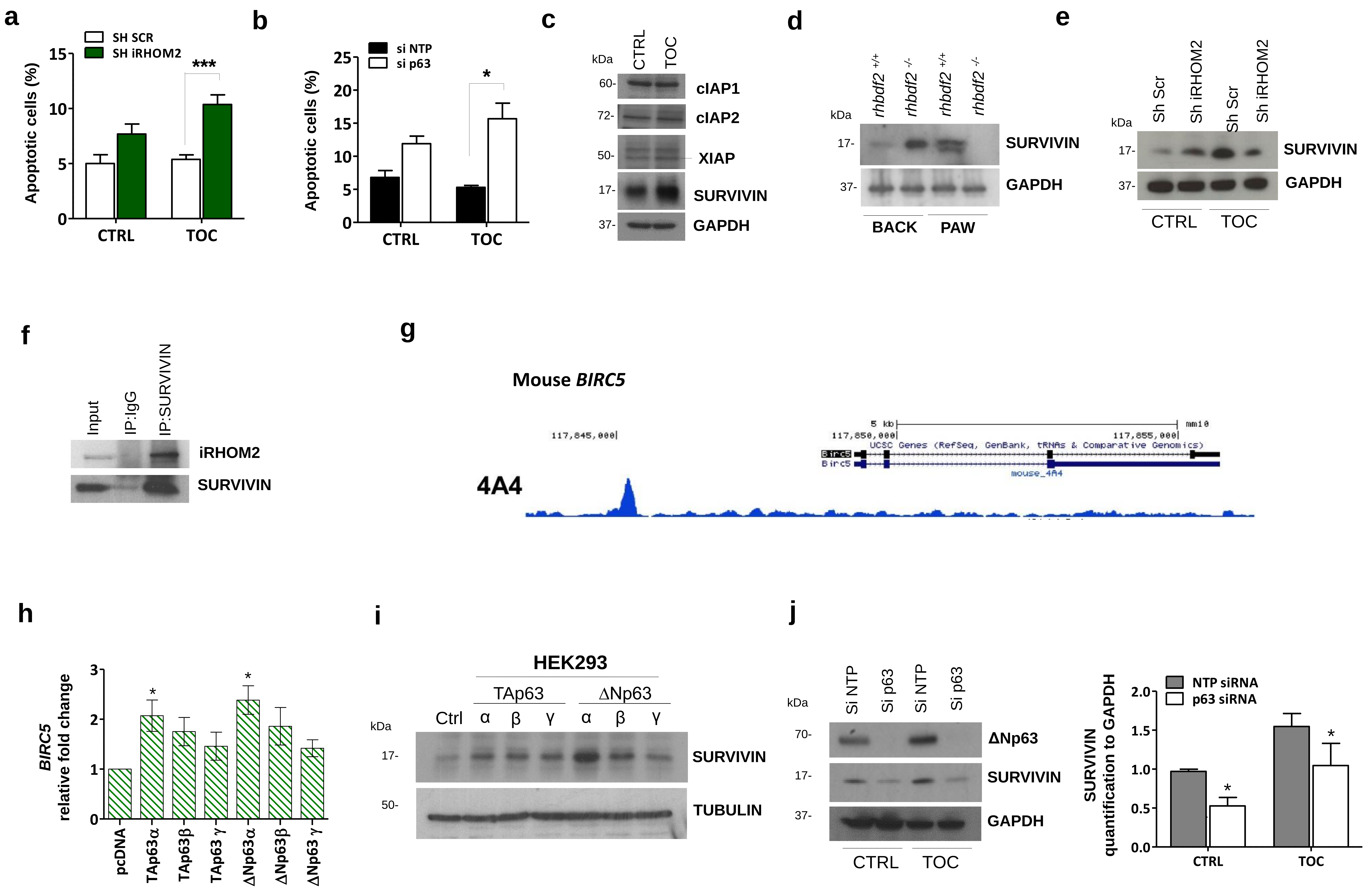
a
SH SCR
SH iRHOM2
Apoptotic cells (%)
***
CTRL
TOC
b
si NTP
si p63
Apoptotic cells (%)
*
CTRL
TOC
c
kDa
CTRL
TOC
cIAP1
cIAP2
XIAP
SURVIVIN
GAPDH
d
rhbdf2 +/+
rhbdf2 -/-
rhbdf2 +/+
rhbdf2 -/-
SURVIVIN
GAPDH
BACK
PAW
e
Sh Scr
Sh iRHOM2
Sh Scr
Sh iRHOM2
SURVIVIN
GAPDH
CTRL
TOC
f
Input
IP:IgG
IP:SURVIVIN
iRHOM2
SURVIVIN
g
Mouse BIRC5
4A4
h
BIRC5 relative fold change
pcDNA
TAp63α
TAp63β
TAp63 γ
ΔNp63α
ΔNp63β
ΔNp63 γ
i
HEK293
TAp63
ΔNp63
Ctrl
α
β
γ
SURVIVIN
TUBULIN
j
Si NTP
Si p63
Si NTP
Si p63
ΔNp63
SURVIVIN
GAPDH
CTRL
TOC
NTP siRNA
p63 siRNA
SURVIVIN quantification to GAPDH

Supplementary Figure 4. iRHOM2-p63 axis modulates resistance to apoptosis. (a) Percentage of apoptotic cells was quantified by using flow cytometric analysis of Annexin-V-positive populations in Sh-Scr and Sh-iRHOM2-transfected CTRL and TOC keratinocytes. **(b)** Percentage of apoptotic cells measured with Annexin V assay by flow cytometry in CTRL and TOC keratinocytes transfected with non-targeting pool (NTP) or p63 siRNA. Data in **(a)** and **(b)** are expressed as mean with SEM from four experiments. Student's t-test shows $p<0.05$ (*) and $p<0.001$ (***). **(c)** Immunoblotting of lysates from CTRL and TOC keratinocytes analysed for cIAP1, cIAP2, XIAP and SURVIVIN. GAPDH is used as a loading control. **(d)** Representative immunoblot for SURVIVIN using lysates taken from back skin or fore-paw of *rhbdf2*$^{+/+}$ and r*hbdf2*$^{-/-}$ mice. GAPDH was used as a loading control. **(e)** Immunoblotting of SURVIVIN expression in CTRL and TOC keratinocytes depleted for iRHOM2. GAPDH was used as loading control. **(f)** CTRL keratinocyte lysates were immunoprecipitated with anti-SURVIVIN and immunoblotted with anti-iRHOM2 antibody. **(g)** Screenshot of the UCSC genome browser from ChIP-seq analysis of normal mouse primary keratinocytes with 4A4 antibody. The p63 binding sites identified in this ChIP-seq study were previously reported. **(h)** qRT-PCR was performed for *BIRC5* in HEK293 cells transfected with TA and ΔNp63 isoforms, 9 hours after transfection. The graph represents means and SEM of three biological replicates. Student's two-tailed t-test was used for statistical evaluation ($p<0.05$ (*)). **(i)** Representative WB for SURVIVIN was performed in HEK293 cells over-expressing p63 isoforms. TUBULIN was used as loading control. **(j)** Immunoblotting for SURVIVIN in CTRL and TOC keratinocytes with NTP and p63 siRNA. GAPDH was used as a loading control. The quantifications included were performed by ImageJ software and compared to the loading control in three independent experiments. Student's t-test shows $p<0.05$ (*).

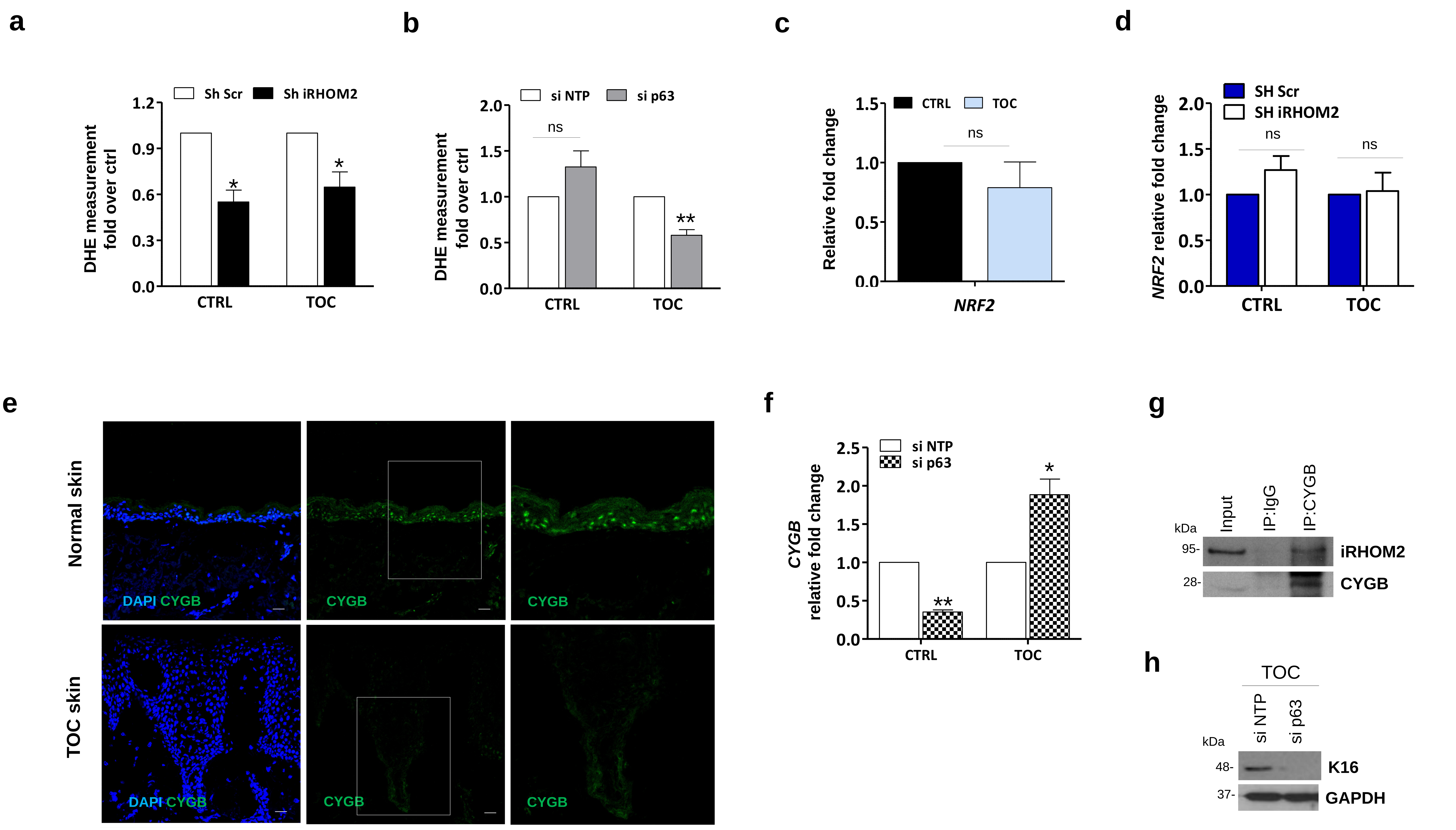
a
Sh Scr
Sh iRHOM2
DHE measurement fold over ctrl
1.2
0.9
0.6
0.3
0.0
*
*
CTRL
TOC
b
si NTP
si p63
DHE measurement fold over ctrl
2.0
1.5
1.0
0.5
0.0
ns
**
CTRL
TOC
c
CTRL
TOC
Relative fold change
1.5
1.0
0.5
0.0
ns
NRF2
d
SH Scr
SH iRHOM2
NRF2 relative fold change
2.0
1.5
1.0
0.5
0.0
ns
ns
CTRL
TOC
e
Normal skin
TOC skin
DAPI CYGB
CYGB
CYGB
DAPI CYGB
CYGB
CYGB
f
si NTP
si p63
CYGB relative fold change
2.5
2.0
1.5
1.0
0.5
0.0
**
*
CTRL
TOC
g
kDa
Input
IP:IgG
IP:CYGB
95-
iRHOM2
28-
CYGB
h
TOC
si NTP
si p63
kDa
48-
K16
37-
GAPDH

Supplementary Figure 5. Repression of CYGB leads to increase oxidative stress in hyperproliferative keratinocytes. (a) Quantification of DHE staining by flow cytometry in CTRL and TOC keratinocytes with iRHOM2 depletion by sh-RNA. **(b)** Quantification of DHE staining by flow cytometry in CTRL and TOC keratinocytes transfected with p63 siRNA. Data in **(a)** and **(b)** are expressed as the mean and SEM of three independent experiments. Student's t-test shows $p<0.05$ (*), $p<0.01$ (**) and no significant difference (ns). **(c-d)** Relative mRNA levels of *NRF2* in control (CTRL) and TOC keratinocytes depleted for iRHOM2. Data represent three independent experiments with similar results. Student's t-test shows no significant difference (ns) between the samples. **(e)** Representative confocal microscopy images from immunostaining of CYGB in normal and TOC skin. DAPI (blue) is used as a nuclear stain. Scale bar: 20 µm. **(f)** mRNA expression by qRT-PCR of *CYGB* in CTRL and TOC cells transfected with NTP or p63 siRNA. The graph represents means and SEM of three biological replicates. Student's t-test shows $p<0.05$ (*), $p<0.01$ (**). **(g)** CTRL keratinocyte lysates were immunoprecipitated with anti-CYGB antibody and immunoblotted with anti-iRHOM2 antibody. **(h)** Representative WB of keratin 16 (K16) in TOC keratinocytes transfected with non-targeting pool (NTP) or p63 siRNA. GAPDH is used as loading control.

Supplementary Fig. 6

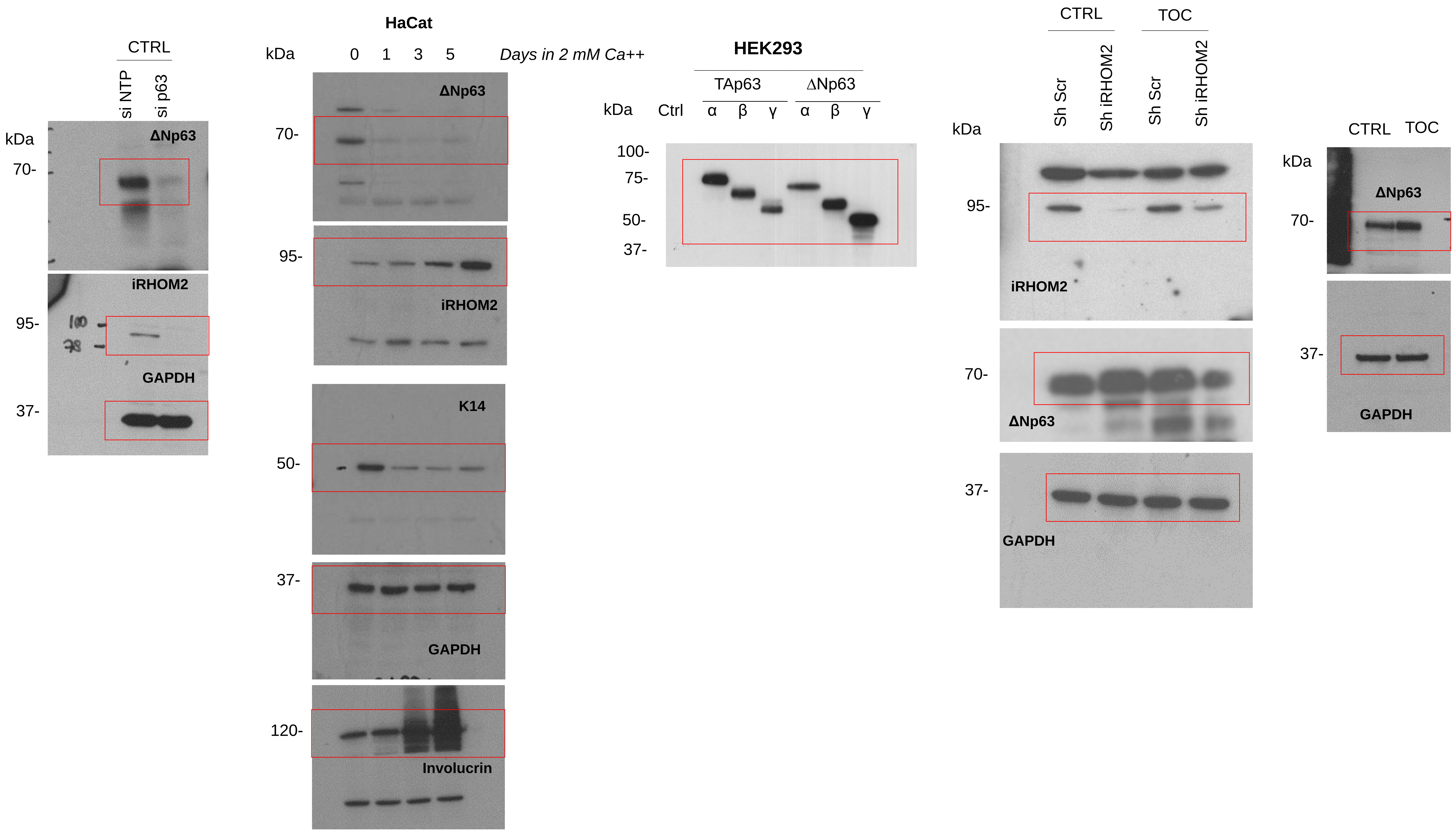

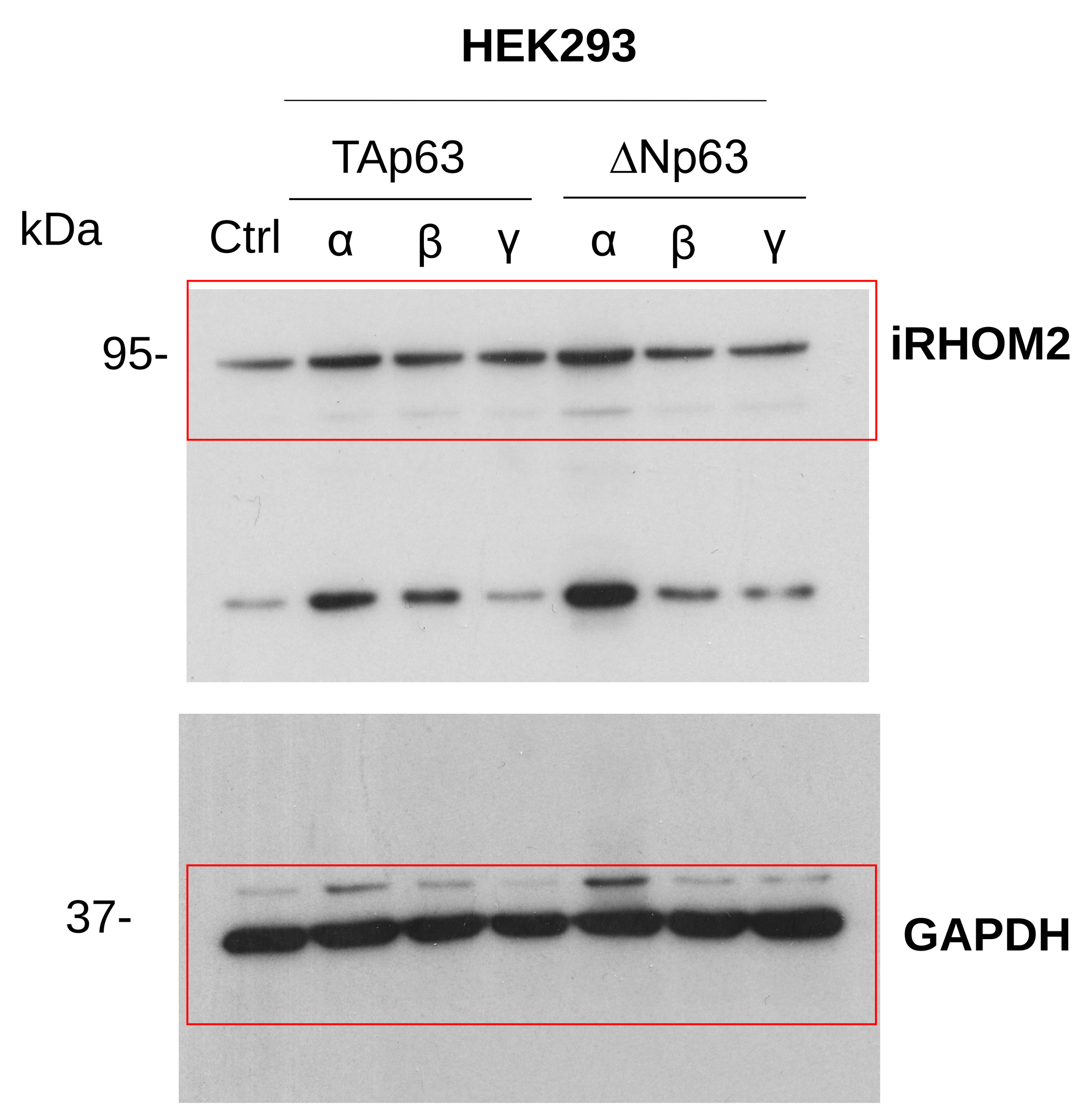
HEK293
TAp63
ΔNp63
kDa
Ctrl α β γ α β γ
95-
iRHOM2
37-
GAPDH

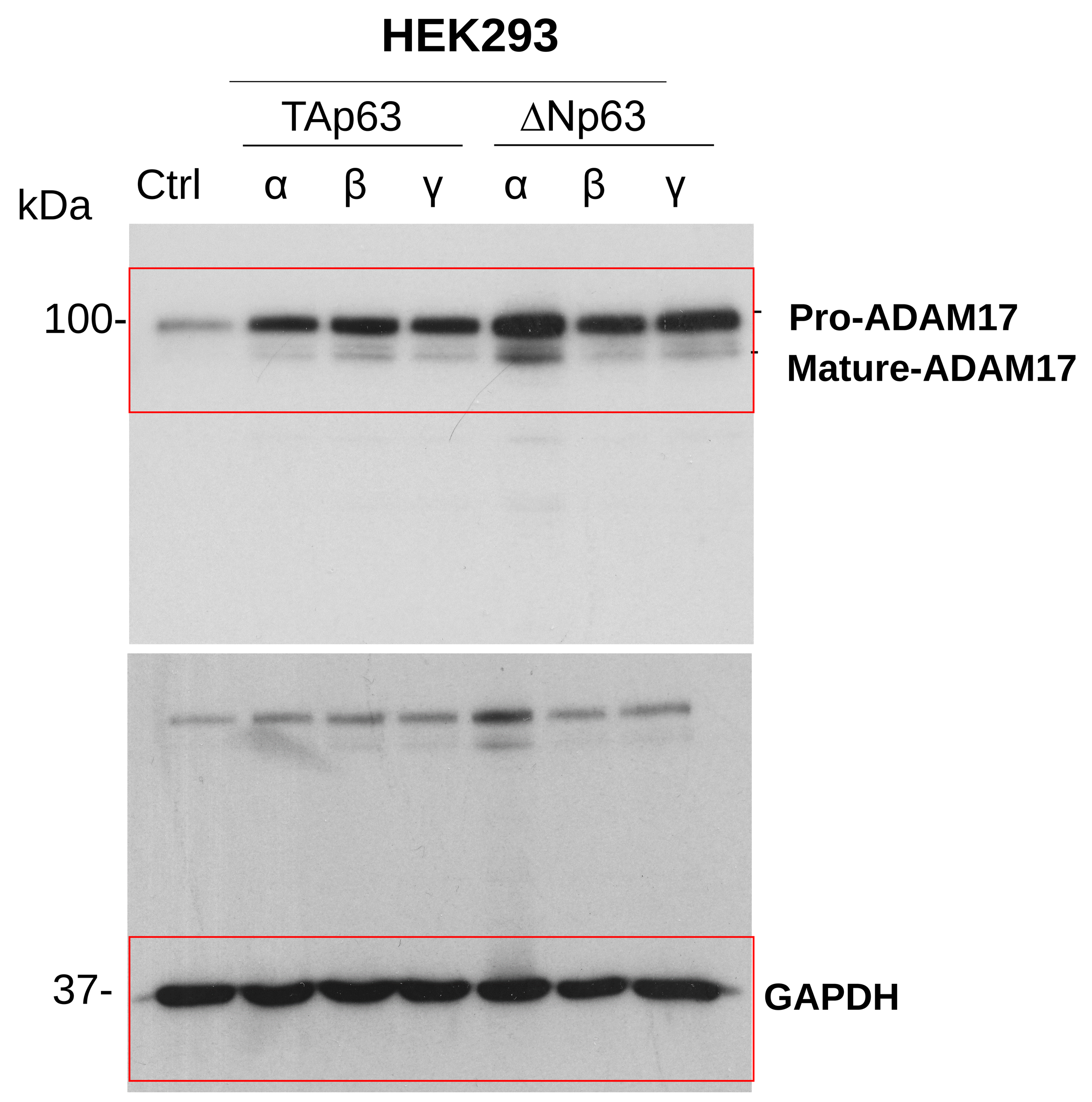
HEK293
TAp63
ΔNp63
kDa
Ctrl α β γ α β γ
100-
Pro-ADAM17
Mature-ADAM17
37-
GAPDH

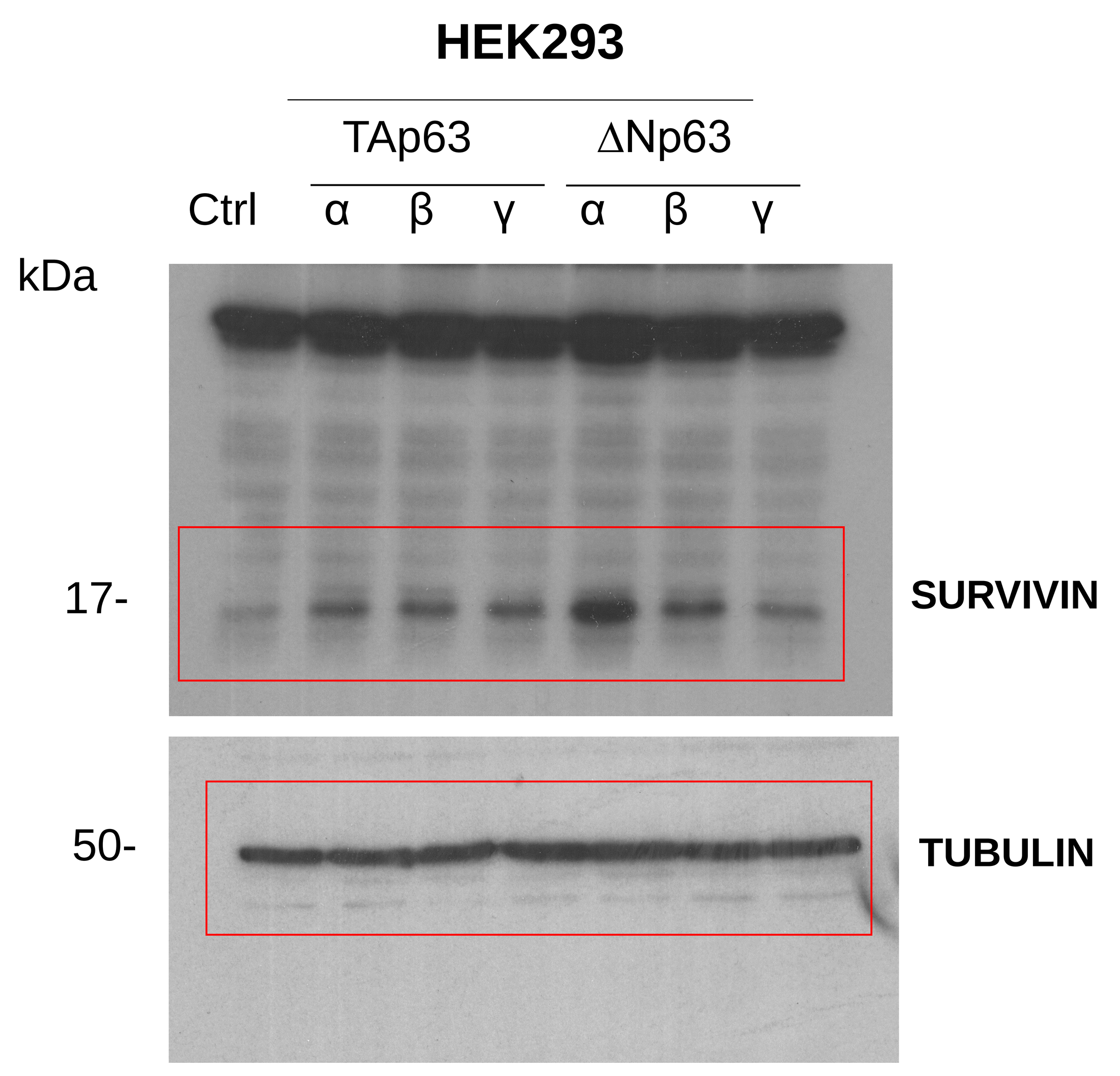
HEK293
TAp63
ΔNp63
Ctrl α β γ α β γ
kDa
17-
SURVIVIN
50-
TUBULIN

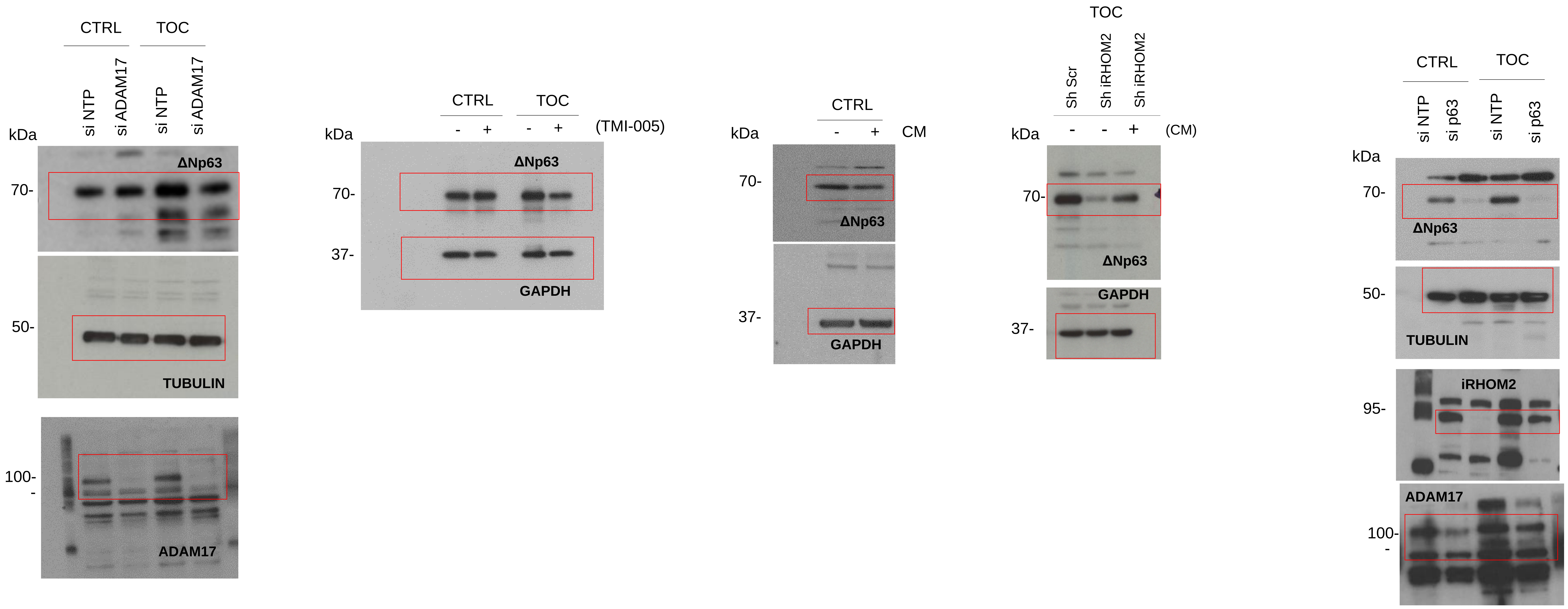
CTRL
TOC
si NTP
si ADAM17
si NTP
si ADAM17
kDa
ΔNp63
70-
50-
TUBULIN
100-
ADAM17
CTRL
TOC
- + - +
(TMI-005)
kDa
ΔNp63
70-
37-
GAPDH
CTRL
- +
CM
kDa
70-
ΔNp63
37-
GAPDH
TOC
Sh Scr
Sh iRHOM2
Sh iRHOM2
kDa
- - +
(CM)
70-
ΔNp63
GAPDH
37-
CTRL
TOC
si NTP
si p63
si NTP
si p63
kDa
70-
ΔNp63
50-
TUBULIN
iRHOM2
95-
ADAM17
100-

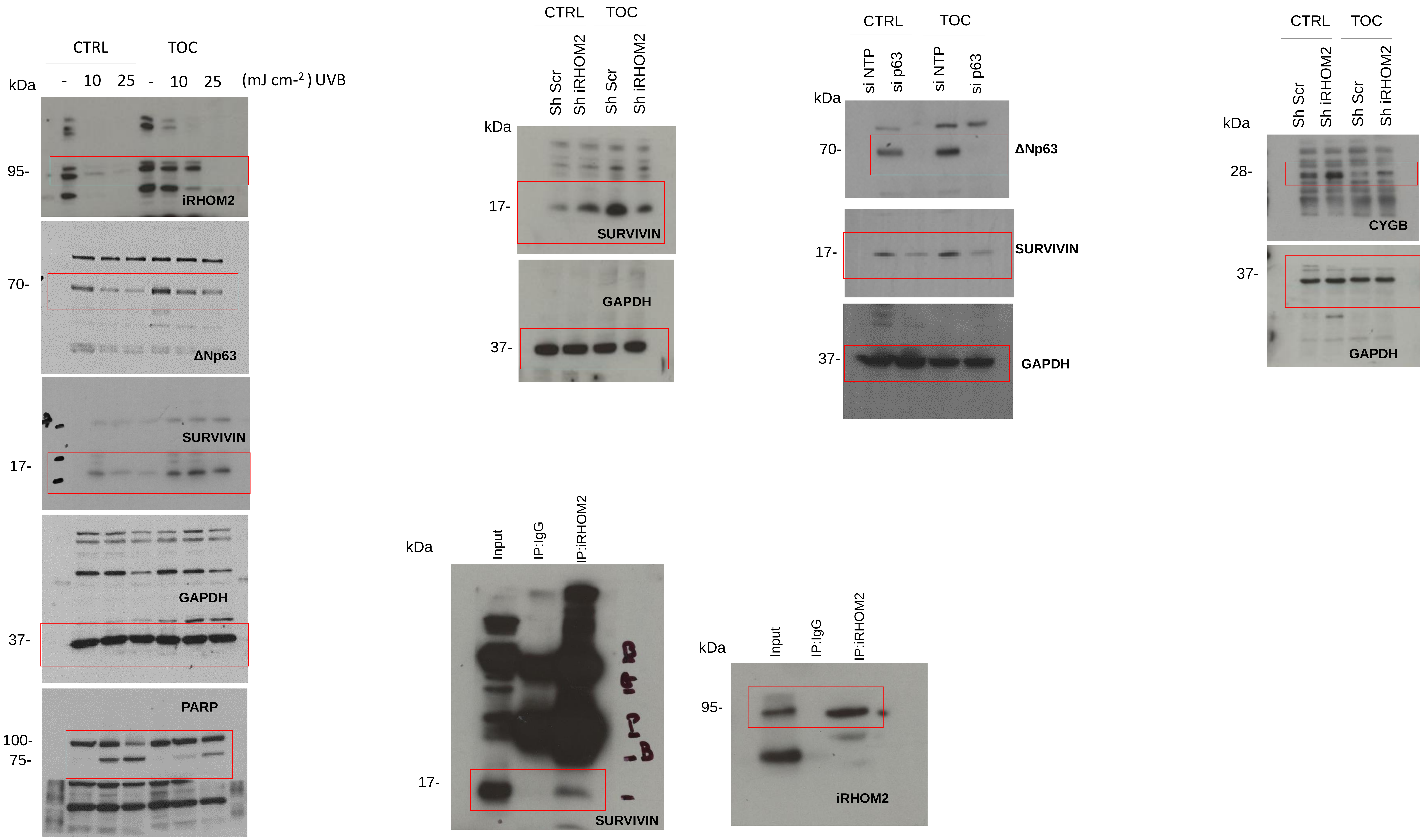
CTRL
TOC
kDa
- 10 25 - 10 25 (mJ cm-2) UVB
95-
iRHOM2
70-
ΔNp63
17-
SURVIVIN
GAPDH
37-
PARP
100-
75-
CTRL TOC
Sh Scr
Sh iRHOM2
Sh Scr
Sh iRHOM2
kDa
17-
SURVIVIN
GAPDH
37-
CTRL TOC
si NTP
si p63
si NTP
si p63
kDa
70-
ΔNp63
17-
SURVIVIN
37-
GAPDH
CTRL TOC
Sh Scr
Sh iRHOM2
Sh Scr
Sh iRHOM2
kDa
28-
CYGB
37-
GAPDH
kDa
Input
IP:IgG
IP:iRHOM2
17-
SURVIVIN
kDa
Input
IP:IgG
IP:iRHOM2
95-
iRHOM2

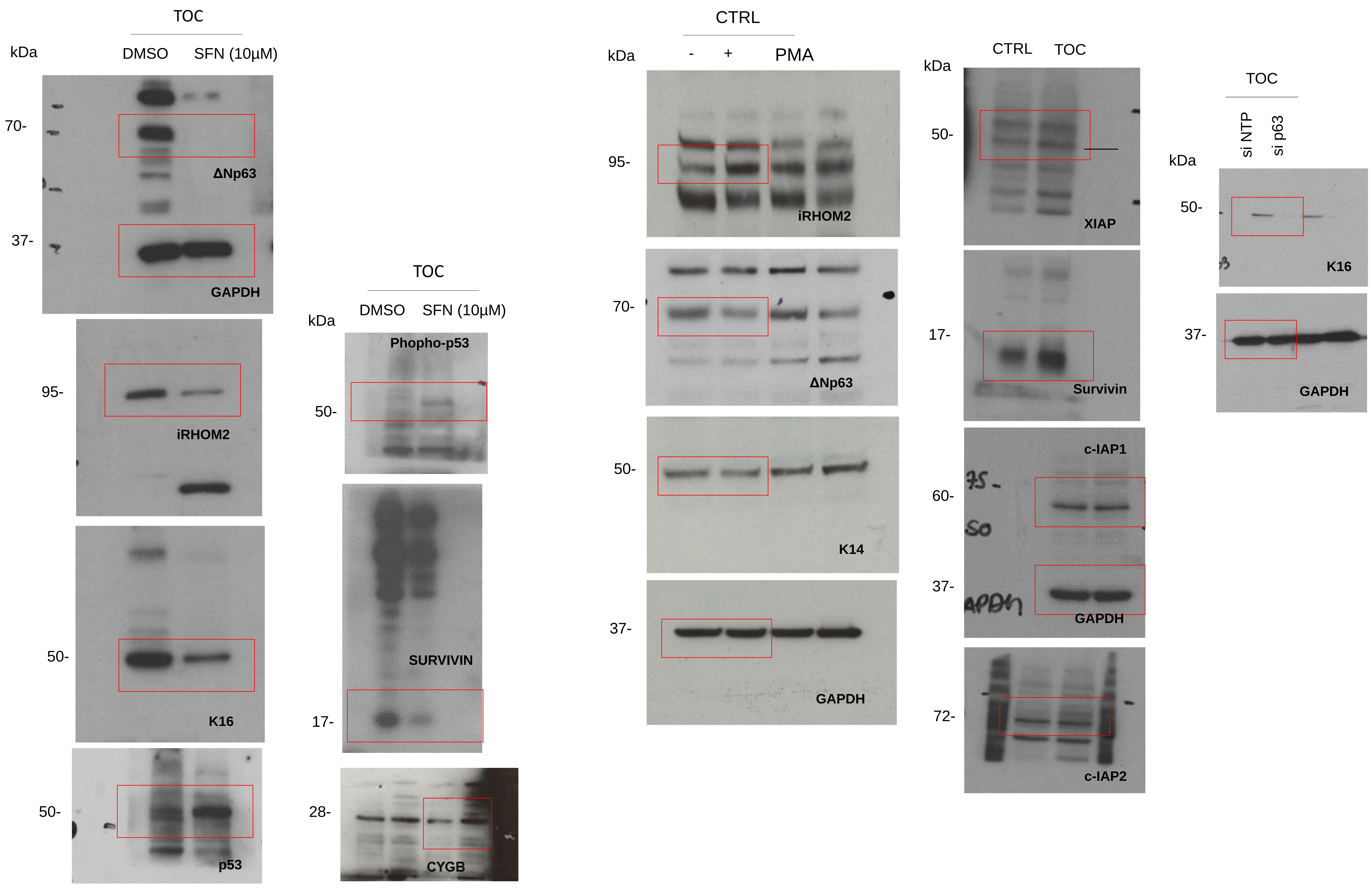

TOC
kDa
DMSO
SFN (10µM)
70-
ΔNp63
37-
GAPDH
95-
iRHOM2
50-
K16
50-
p53
TOC
kDa
DMSO
SFN (10µM)
Phopho-p53
50-
SURVIVIN
17-
28-
CYGB
CTRL
kDa
-
+
PMA
95-
iRHOM2
70-
ΔNp63
50-
K14
37-
GAPDH
kDa
CTRL
TOC
50-
XIAP
17-
Survivin
c-IAP1
60-
37-
GAPDH
72-
c-IAP2
TOC
si NTP
si p63
kDa
50-
K16
37-
GAPDH

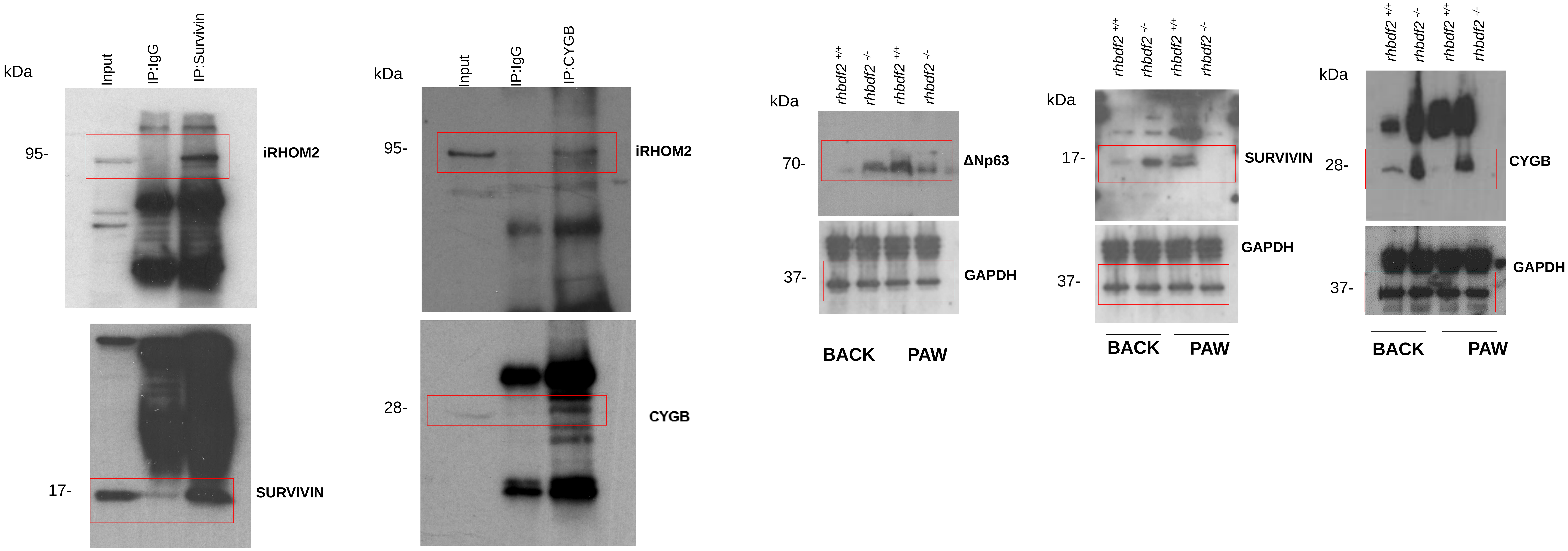

Supplementary Figure 6. Uncropped images of western blots.

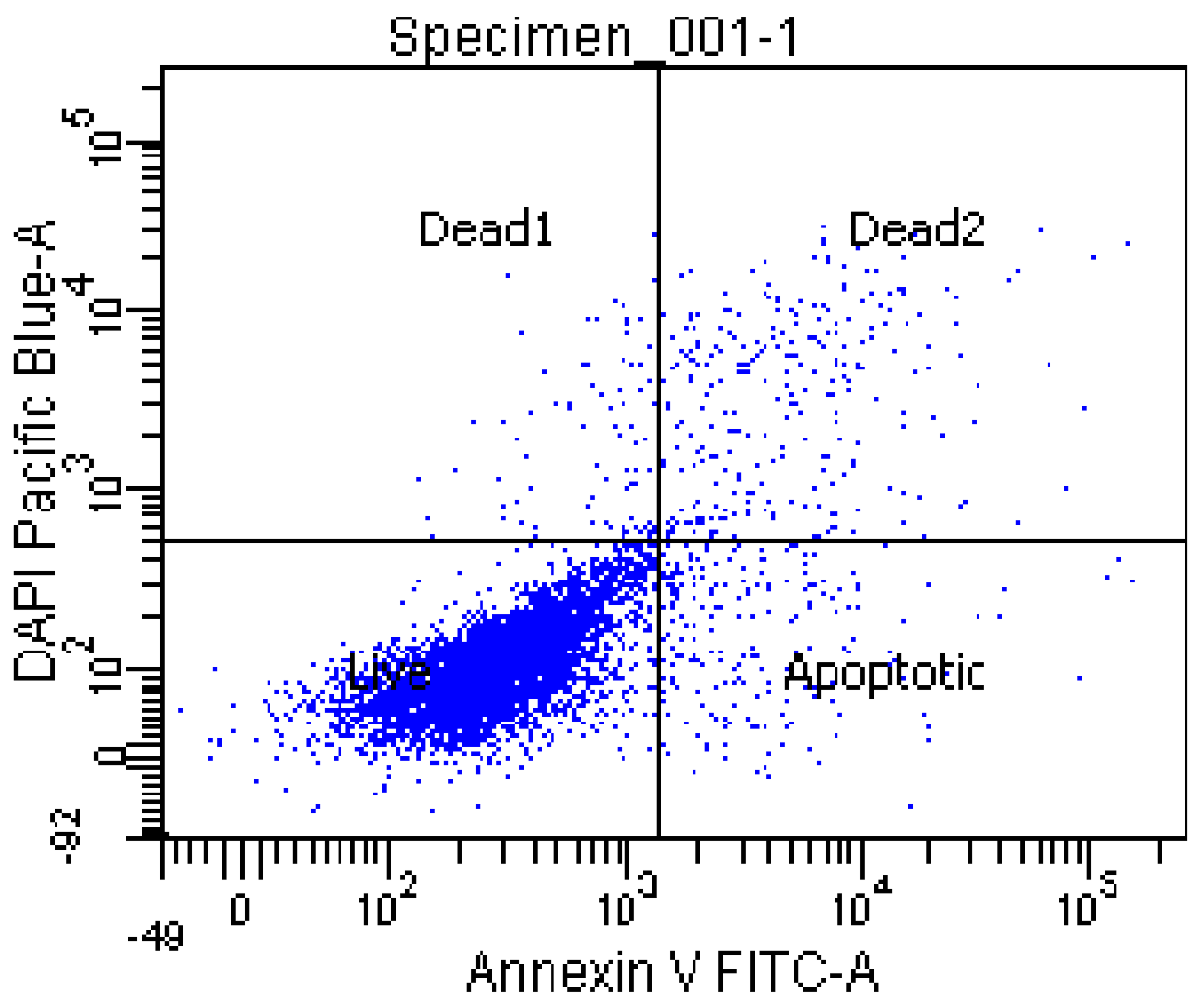

Supplementary Figure 7. Cells were analysed with FACS Diva 6.1.3 software at an excitation with 488 nm laser and emission at 530 nm for FITC and with 405 nm laser and emission at 460 nm for DAPI. A minimum of 20,000 cells was analysed per sample, as illustrated the cells were gated on a dot-plot of annexin V-FITC vs DAPI with a quadrant placed marking live cells in the double negative quadrant (annexin V negative, lower left), and with annexin V positive indicated by Early Apoptotic (low expression of annexin V, lower right) and Late Apoptotic (annexin V positive, upper right).

Supplementary Table 1. List of used antibodies

Target	Clone	P/M	Notes	Source	WB dilution	ICC/IHC dilution	ChIP
Adam17 (Rabbit)		Polyclonal		ab2051 (Abcam)	1:500	X	X
K6 (Mouse)	KA12	Monoclonal		ab18586 (Abcam)	X	1:100	X
K16 (Mouse)	LL025	Monoclonal		ab8741 (Abcam)	1:500	X	X
K16 (Rabbit)	EP1615Y	Monoclonal		ab76416 (Abcam)	X	1:100	X
c-IAP1 (Rabbit)		Polyclonal		ab2399 (Abcam)	1:1000	X	X
c-IAP2 (Rabbit)		Polyclonal		ab137393 (Abcam)	1:1000	X	X
α-Tubulin (Rabbit)		Polyclonal		ab4074 (Abcam)	1:2500	X	X
GAPDH (Rabbit)		Polyclonal		ab9485 (Abcam)	1:2500	X	X
p63 (Rabbit)		Polyclonal	H137 α pan-p63	sc-8343 (Santa Cruz)	X	1:100	2μg
p53 (Mouse)	DO-1	Monoclonal		sc-126 (Santa Cruz)	1:500	X	X
CYGB (Mouse)	D-7	Monoclonal		sc-365246 (Santa Cruz)	X	1:50	X
K14 (Mouse)	LL001	Monoclonal		sc-53253 (Santa Cruz)	X	1:100	X
XIAP (1/500,		Polyclonal		sc-11426 (Santa Cruz)	1:500	X	X
PARP (Rabbit)		Polyclonal		9542 (Cell Signaling)	1:500	X	X
phospho-p53 Ser15 (Rabbit)		Polyclonal		92845 (Cell Signaling)	1:500	X	X
ΔNp63 (Rabbit)		Polyclonal		619001 (BioLegend)	1:500	X	X
iRHOM2 (Rabbit)		Polyclonal		SAB1304414 (Sigma)	1:500	1:100	X
Involucrin (Rabbit)		Polyclonal		SAB4501594 (Sigma)	1:1000	X	X
K14 (mouse)	LL002	Monoclonal		(Cancer Research UK)	1:500	X	X
CYGB (Rabbit)		Polyclonal		HPA017757 (Sigma)	1:350	X	X
Survivin (Rabbit)		Polyclonal		NB500-201 (Novus)	1:500	1:100	X
IgG (Rabbit)		Polyclonal		Sc-2027 (Santa Cruz)	X	X	2μg
